# Environmental Impact of Waste Treatment and Synchronous Hydrogen Production: Based on Life Cycle Assessment Method

**DOI:** 10.3390/toxics12090652

**Published:** 2024-09-05

**Authors:** Yiting Luo, Rongkui Su

**Affiliations:** 1 School of Business, Hunan First Normal University, Changsha 410114, China; yitingl2021@163.com; 2 National Engineering Laboratory of Southern Forestry Ecological Application Technology, Changsha 410004, China; 3 PowerChina Zhongnan Engineering Corporation Limited, Changsha 410004, China; 4 College of Life and Environmental Sciences, Central South University of Forestry and Technology, Changsha 410004, China

**Keywords:** environmental waste, synchronous hydrogen production, LCA, ecological impact, sustainable development

## Abstract

Based on the life cycle assessment methodology, this study systematically analyzes the energy utilization of environmental waste through photocatalytic treatment and simultaneous hydrogen production. Using 10,000 tons of organic wastewater as the functional unit, the study evaluates the material consumption, energy utilization, and environmental impact potential of the photocatalytic waste synchronous hydrogen production system (specifically, the synchronous hydrogen production process of 4-NP wastewater with CDs/CdS/CNU). The findings indicate that potential environmental impacts from the photochemical treatment of environmental waste and synchronous hydrogen production primarily manifest in freshwater ecological toxicity, marine ecological toxicity, terrestrial ecological toxicity, and non-carcinogenic toxicity to humans. These ecological impacts stem from the catalyst’s adsorption and metal leaching during the photo-degradation and hydrogen production processes of environmental waste. By implementing reasonable modifications and morphological refinements to the catalyst, these effects can be mitigated while achieving enhanced efficiency in environmental waste processing and simultaneous hydrogen production. The research outcomes provide valuable insights for advancing sustainable development in green technology for environmental waste treatment and energy utilization.

## 1. Introduction

With the ongoing advancement of national industrialization and urbanization, the issues of energy scarcity and environmental degradation have gained increasing prominence. Energy resources, particularly strategic ones, are crucial for ensuring both livelihoods and economic development. However, the escalating environmental impacts resulting from the excessive exploitation and utilization of energy resources profoundly affect human life quality and economic and societal sustainability [1,2]. Waste is defined as material that loses its utility within specific temporal and spatial contexts due to excessive consumption in daily life and societal activities, resulting in its environmental accumulation [3,4,5,6,7]. The term “energy conversion of environmental waste” refers to the process of utilizing techniques such as biogas production, incineration, and plasma gasification to convert environmental waste into a form of energy that can be reused. The ultimate objective is to achieve comprehensive and efficient utilization of environmental waste [8]. From 2005 to 2030, global aggregate energy demand is projected to increase significantly by 50% [9]. Depletion of fossil fuels and concerns regarding energy sustainability have prompted global efforts towards developing renewable energy sources [10]. To address the escalating energy demand, various countries have successfully implemented waste-to-energy processes [11,12]. By 2014, waste contributed significantly to global power generation, providing 93,540 gigawatt-hours of electricity (0.4% of the world’s total) and 399,734 terajoules of heat (equivalent to 2.7% of the world’s total power generation) [13].

The primary forms of utilizing environmental waste as energy sources include the utilization through synthesis gas, solid fuels, pyrolysis oil, charcoal, biogas, and bioethanol [14,15,16,17]. Among these, the utilization of waste incineration for power generation and biogas heating are the two most prevalent forms [18]. However, these conventional methods of utilizing waste material for energy conversion may potentially give rise to secondary pollution issues [19,20,21]. Environmentally friendly practices and sustainable development constitute the inevitable trajectory towards the conversion of environmental waste into energy [22]. In the current landscape of commercial energy, 95% comprises fossil fuels whose extensive utilization engenders climate predicaments at a global scale, exemplified by the emission of NO_x_, SO_2_, CO_2_, and their ilk [23,24]. Therefore, an increasing number of researchers are striving to develop technological processes that simultaneously degrade environmental pollutants and produce clean energy, in order to address the current challenges of resource scarcity and environmental degradation [25].

Countries around the world are actively researching renewable alternative energy sources such as hydrogen energy, biomass energy, ocean energy, geothermal energy, and solar energy [26,27]. Among these, hydrogen energy has become one of the hottest new energies in current research due to its renewable nature and the fact that it does not generate pollutants during combustion or afterwards [28,29]. The technology of synchronous hydrogen production through waste disposal refers to the utilization of waste in order to produce hydrogen, thereby achieving an inexpensive acquisition of hydrogen energy while also allowing for the resource utilization of waste materials [30]. The photocatalytic degradation of organic pollutants with simultaneous hydrogen generation commenced in the early 1990s, during the previous century [31]. Organic pollutants present in wastewater can be utilized as sacrificial agents, facilitating photocatalytic water splitting for hydrogen production [32]. This approach not only enables the efficient conversion of pollutants and recycling of water resources but also effectively mitigates environmental contamination caused by wastewater [33].

The key to the photocatalytic degradation of organic pollutants with simultaneous hydrogen generation lies in the development of synthetic, stable and environmentally friendly photocatalytic materials to achieve the simultaneous objectives of degradation and hydrogen production through photo-induced reduction [34,35,36,37,38]. Photocatalytic technology, harnessing the power of the sun, is widely regarded as the paramount method for pollution reduction and hydrogen production, capturing the unwavering attention of numerous nations [39]. For example, the ternary photocatalytic material (CDs/CdS/CNU) was prepared by combining layered materials (CN-U) with metal chalcogenides (CdS) and loading carbon quantum dots (CDs) with good light absorption and upconversion effects. Then, the results indicate that the synthesized composite photocatalysts can efficiently treat organic wastewater (4-NP) and simultaneously produce hydrogen [40,41]. However, there has been a lot of research and development on the technology of photocatalytic degradation of pollutants and simultaneous hydrogen production, but there is relatively little research on its environmental impact.

In order to systematically evaluate the environmental impact of photocatalytic pollutant treatment and simultaneous hydrogen production, this study takes the application case of CD/CDs/CNU in the simultaneous hydrogen production of 4-NP containing wastewater as the research object and systematically evaluates its potential environmental risks through the life cycle assessment (LCA) method. Life cycle assessment (LCA) is a comprehensive method for assessing environmental impacts, adhering to the principle of “cradle to grave”, which can ensure the credibility and scientific rigor of assessing environmental impacts related to waste energy utilization [16,42,43]. The work carried out in this study includes the following: (1) the research objectives and scope of photocatalytic waste treatment and synchronous hydrogen production are defined; (2) the full life cycle inventory of photocatalytic waste treatment and synchronous hydrogen production was investigated and analyzed; (3) based on the LCA method, the ecological and environmental impact and potential value of photocatalytic waste treatment and synchronous hydrogen production were comprehensively evaluated; (4) we comprehensively analyzed the environmental hazards of photocatalytic waste treatment and synchronous hydrogen production and gave targeted improvement measures. This study will provide theoretical support for the practical application of photocatalytic waste treatment and synchronous hydrogen production and provide some reference for the green development of environmental waste energy utilization technology.

## 2. Materials and Methods

### 2.1. The Principles of the Life Cycle Methods

With the continuous progress of human civilization, the escalating devastation of the global ecological environment has become increasingly apparent. The conventional methods of managing and remedying environmental issues are no longer applicable to the diverse array of challenges we face today. Therefore, embracing an environmental management approach based on the concept of life cycle thinking is much more conducive to achieving a harmonious and sustainable balance between the economy and the environment [44]. Life cycle assessment (LCA), as an environmental management tool for product systems, has gradually gained acceptance and application in various domains, regarded as the most promising environmental management tool of the 21st century [45,46]. The International Organization for Standardization (ISO), in its ISO 14040 standard, defines life cycle assessment as the quantitative and qualitative analysis and evaluation of the resource consumption and waste emissions caused by a product throughout its life cycle [47].

This study adheres to the boundary demarcation and data processing standards outlined in ISO 14040 and GB/T 24040. We utilize the ReCiPe method for calculations, which is widely employed in life cycle assessment (LCA) to analyze and quantify the environmental impacts of a product across its entire life cycle, from raw material acquisition to production, use, and disposal [1,2]. It focuses on midpoint (problem-oriented) and endpoint (damage-oriented) impact categories. ReCiPe not only assesses traditional environmental impacts such as acidification, eutrophication, and ecosystem toxicity, resource consumption and climate change impacts are also included, providing a more comprehensive assessment perspective. ReCiPe data sources are categorized into foreground and background processes. The foreground processes are derived from our field investigations and address specific aspects that may vary from other studies or result from technological advancements. Background processes build upon comprehensive LCA data from existing research. In this study, data from background processes are clearly cited in the manuscript, while foreground process data, which do not have external citations, originate from our own research.

The life cycle approach evaluates the “inputs” and “outputs” of each process [48]. According to ISO 14040, we will have certain trade-offs in the process of discussion and traceability, as long as the corresponding trade-off rules are met; otherwise, the traceability will be endless. The regulations also point out that there are two types of LCA that do not need to trace the process. One is natural resources; we only need to know the consumption of natural resources and do not need to trace their production process. The other is environmental emissions (including greenhouse gasses, atmospheric/water/soil pollutants, solid waste, etc.); again, we only need to know the discharge and do not need to know the natural process after discharge.

### 2.2. The Fundamental Analytical Framework of the Life Cycle Approach

**Figure 1:**
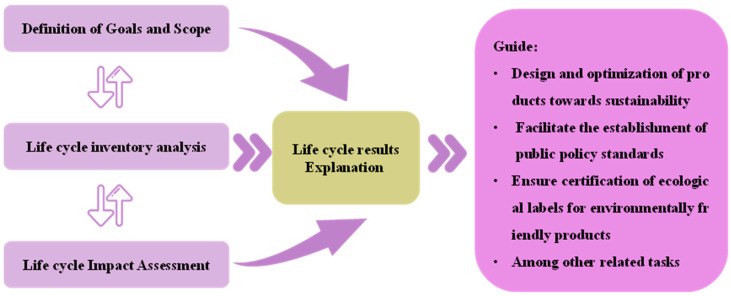
Life cycle evaluation framework diagram.

According to ISO 14040 and GB/T 24040, the entire process of life cycle assessment can be divided into four main components, namely the definition of objectives and scope, analysis of the life cycle inventory, evaluation of the life cycle impacts, and interpretation of the life cycle results [47,49,50]. The relationship between these components is depicted in Figure 1.

(1) Definition of the goal and the scope

The primary component of LCA research is the determination of evaluation objectives. The definition of functional units and system boundaries associated with these objectives guides the analysis structure, ultimately providing an assessment of environmental impact types. In this chapter, the evaluation system’s objectives and scope are meticulously divided based on the varying technologies for environmental waste energy conversion.

(2) Life cycle inventory analysis

The term “life cycle inventory analysis” refers to the identification of the subject of study, wherein data pertaining to all energy conversion processes involved from the origin to the final destination are collected and computed. The resulting energy input and output data constitute a comprehensive inventory of the life cycle evaluation system, which serves as a research reference for analyzing issues during the product production transformation process.

(3) Impact evaluation

The assessment of the environmental impacts in LCA is a crucial step, encompassing method selection, impact categorization analysis, and impact calculation process. It ultimately relies on scientifically grounded evaluations derived from the integration of mathematical models.

(4) The result interpretation

The outcome interpretation marks the final stage in the LCA analysis. Through the aforementioned three steps, it establishes the identity of the research analysis subject, defines the system boundaries for inclusion in the inventory, and incorporates model analysis to assess environmental burdens during impact assessment. It provides feasible recommendations, serving to adjust variables within the system framework and rectify adverse causes of environmental impacts.

### 2.3. Data Sources

The data presented in this summary are primarily sourced from the Chinese Life Cycle Inventory Database and relevant literature (specific data sources detailed in Table 1).

## 3. Results and Discussion

### 3.1. The Basic Framework of the LCA Model for Synchronous Hydrogen Production and Environmental Waste Treatment

(1) Definition of the goal and the scope

**Figure 2:**
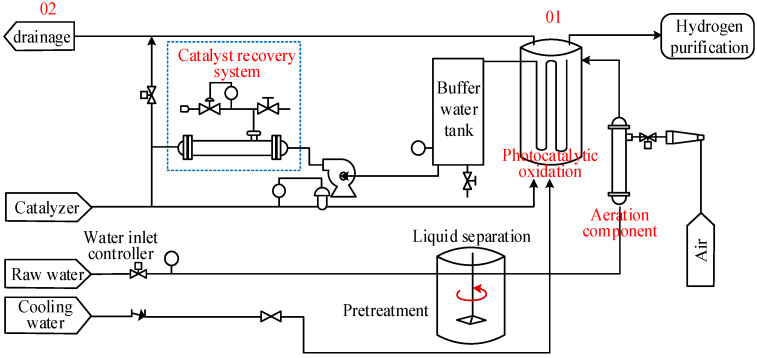
Process diagram for waste treatment and synchronous hydrogen production.

In this section, our study focuses on the integrated hydrogen production system via the photocatalytic treatment of waste materials. Figure 2 illustrates the process flow. The process flow of waste treatment and synchronous hydrogen production includes wastewater collection and transportation, wastewater pretreatment, photocatalyst preparation, wastewater treatment, photocatalytic hydrogen production, catalyst recovery, hydrogen purification, and treated gas and water discharge. In this process, detection points are positioned at sites O1 and O2 to evaluate the influence of environmental indicators on assessing a synchronous hydrogen production system used for catalytic waste treatment. We have adopted a functional unit capable of treating 10,000 metric tons of organic wastewater. Employing a life cycle assessment (LCA) approach, our objective is to analyze resource consumption, energy utilization, and potential environmental impacts related to the photocatalytic treatment of waste materials and the integrated hydrogen production system.

**Figure 3:**
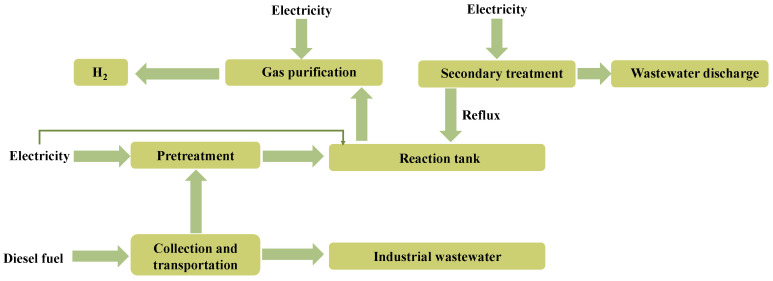
A schematic diagram of the boundary demarcation.

Figure 3 illustrates a diagram depicting the delineation of boundaries. In this study, the life cycle of wastewater treatment and synchronous hydrogen production is divided into the following stages: wastewater collection and transportation stage, wastewater pretreatment stage, photocatalyst preparation stage, wastewater treatment stage, photocatalytic hydrogen production stage, and treated water and gas discharge stage.

(2) Inventory analysis

Table 1 outlines the input–output inventories of each component unit. Key metrics including water quality, waste discharge, equipment operation, and hydrogen production were quantitatively assessed. Electrical energy consumption was computed following the method outlined in the preceding section, and greenhouse gas emissions were evaluated using the same approach. The measured data were chosen to accurately depict wastewater treatment efficacy and its genuine environmental impacts (Table 2).

**Table 1:** Annual life cycle inventory of materials for each functional unit in the photocatalytic treatment and simultaneous hydrogen production process.

Material Input			Material Output		
Wastewater Collection and Transportation Stage	Unit	Magnitude	Photocatalytic Hydrogen Production Stage	Unit	Magnitude
Wastewater [51]	t	10,000	Annual hydrogen production [40,41]	m^3^	5264
Wastewater transportation [52]	km	50	**Gas emission in photocatalytic process (for CDs/CdS/treatment of 4-NP wastewater with CNU)** [53,54]	**Unit**	**Magnitude**
Wastewater transportation fuel consumption [52]	L·a^−1^	13,800			
**Wastewater pretreatment stage**	**Unit**	**Magnitude**	CO_2_	g·m^−3^	9450
Fan power consumption (4 units) [51]	kWh·a^−1^	3160	SO_2_	g·m^−3^	50
Solid–liquid separator power consumption [55]	kWh·a^−1^	3800	N_2_O	g·m^−3^	0
**Photocatalyst preparation stage**	**Unit**	**Magnitude**	CO	g·m^−3^	5.3
Catalyst dosage	kg	2000	NO_x_	g·m^−3^	0.3
Power consumption for CDs/CdS/CNU preparation [40]	kWh·a^−1^	8.0 × 10^4^	VOC	g·m^−3^	0.02
**Wastewater treatment stage**	**Unit**	**Magnitude**	**Content of contaminated elements** [56]	**Unit**	**Magnitude**
CDs/CdS/CNU treatment containing 4-NP wastewater [40]	kWh·a^−1^	6.0 × 10^4^	TN	g/kg	348.2
Power consumption purification by voltage washing in hydrogen purification stage [57]	kWh·a^−1^	2.0 × 10^5^	TP	g/kg	0

a. Wastewater transportation: Primarily carried out through the utilization of diesel engines, with an estimated total fuel consumption of approximately 13,800 L·a^−1^ during the transportation phase.

b. Pre-processing phase: Configure four Granfu wastewater lift pumps. Activate two units for eight hours during the day and activate two units for sixteen hours during the night. The air in the sand removal tank is supplied by a magnetic levitation fan, which is shared with the aerating fan in the aerobic tank. The power consumption of the fan is 3.16 × 10^3^ kWh. The power consumption of the solid–liquid separator is 3.8 × 10^3^ kWh. Therefore, the daily power consumption of the pre-treatment stage’s process equipment is approximately 6.96 × 10^3^ kWh (for processing ten thousand tons of sewage).

c. Cost of photocatalyst preparation: Approximately 2000 kg of photocatalytic material is used for treating 10,000 tons of wastewater. Taking into account the lifespan and manufacturing process of the catalyst, using CDs/CdS/CNU as an example, the electricity required to prepare one year’s worth of photocatalyst is approximately 8.0 × 10^4^ kWh.

d. Wastewater treatment in reaction tanks: The total annual electricity consumption of the circulating agitator is 6.0104 kilowatt-hours. The annual consumption of 4-NP wastewater treatment using the solid–liquid separation machine CDs/CdS/CNU, after catalyst dehydration, is quantified at 4.0 × 10^5^ kWh. The photocatalyst employed in this process harnesses solar irradiation, rendering any external light source unnecessary.

e. Hydrogen purification phase: An elevated hydraulic pressure is employed to eliminate impurities from the hydrogen, while adhering to an annual electricity consumption target denoted by 2.0 × 10^5^ kWh·a^−1^. The annual quantity of purified hydrogen amounts to approximately 5264 cubic meters. The comprehensive energy consumption, as well as the data when converted to coal equivalent, is presented below:

**Table 2:** Total energy consumption and coal conversion (synchronous hydrogen production process of 4-NP wastewater with CDs/CdS/CNU).

Waste Disposal and Synchronous Hydrogen Production	Wastewater Transportation (L·a^−1^)	Pretreatment (kWh·a^−1^)	Photocatalytic Preparation (kWh·a^−1^)	Reaction Pool Processing (kWh·a^−1^)	Hydrogen Purification (kWh·a^−1^)
Energy consumption	13,800	6.96 × 10^3^	8.0 × 10^4^	4.0 × 10^5^	2.0 × 10^5^
Coal conversion coefficient	6.12 × 10^−4^	3.09 × 10^−3^	1.23 × 10^−4^	2.35 × 10^−4^	1.23 × 10^−4^
Coal conversion	16.89	8.53	9.84	46.87	24.60

### 3.2. Analysis of the Potential Environmental Impact

**Figure 4:**
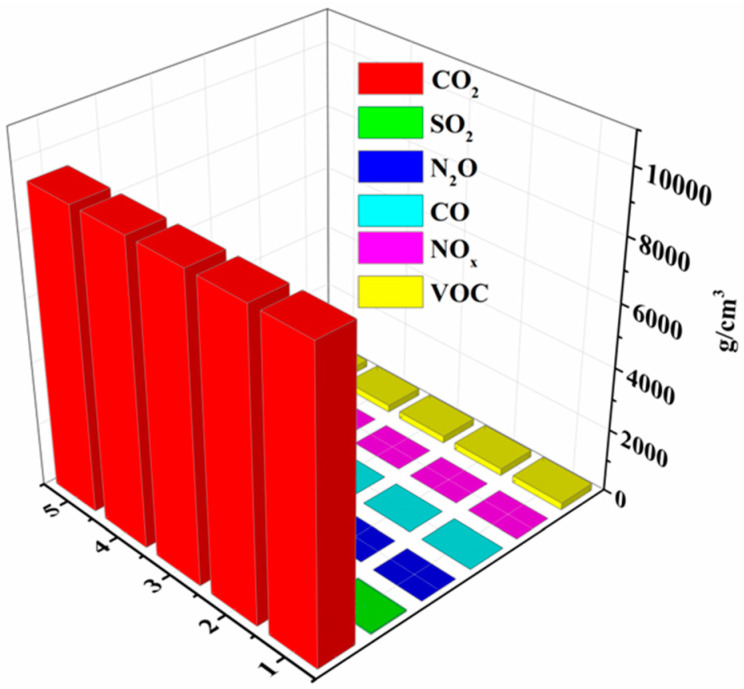
Graph for the detection of other gas emissions during the synchronous hydrogen generation process for treating 4-NP wastewater by using CDs/CdS/CNU.

(1) Determining the types of impact:

During the photolytic treatment of environmental waste and the synchronous hydrogen production process, the degradation of waste and the emission of other gasses into the environment, such as CO_2_, SO_2_, and VOCs, occur during engine and diesel engine operations. Data collection is conducted through five instances of measurements to obtain an average value and ensure the reliability of the data. The specific data regarding the emission of other gasses during the simultaneous hydrogen production process using photosensitizing agents CDs/CdS/CNU for 4-NP wastewater treatment can be observed in Table 3 and Figure 4. It is noteworthy that in the process of treating 4-NP wastewater and simultaneous hydrogen production with photosensitizing agents CDs/CdS/CNU, the generation of CO_2_ is maximal, with negligible emissions of other gasses such as SO_2_, N_2_O, CO, and VOC. Furthermore, it should be emphasized that NO_x_ emerges as the primary environmental contaminant produced within the wastewater.

Furthermore, this section provides a comprehensive analysis of the environmental impacts caused by the photolytic treatment and simultaneous hydrogen production processes of environmental waste. These effects include global warming, stratospheric ozone depletion, ionizing radiation, human health implications, particulate matter formation, terrestrial pollution, land acidification, eutrophication of freshwater bodies, eutrophication of marine ecosystems, toxicity to terrestrial ecosystems, toxicity to freshwater ecosystems, toxicity to marine organisms, carcinogenic toxicity to humans, non-carcinogenic toxic effects on humans, land utilization issues, and scarcity of mineral and fossil resources and water consumption.

(2) Quantifying Standardized Environmental Impact Metrics:

Employing the ReCiPe model [58,59,60], we calculate the characterization factors of the features to shed light on distinct types of environmental impacts. The purpose of characterizing calculations is to convert different substances from each type of environmental impact into a unified parameter for subsequent weighted calculations. ReCiPe 2016 is a general and mature full life cycle environmental impact assessment model with very mature and comprehensive assumptions, groupings, and databases. The ReCiPe model integrates 18 types of potential environmental impacts, including global warming, stratospheric ozone depletion, ionizing radiation, ozone formation and human health, fine particulate matter formation, land acidification, ozone formation, terrestrial ecosystems, freshwater eutrophication, marine eutrophication, terrestrial ecotoxicity, marine ecotoxicity, freshwater ecotoxicity, human carcinogenic toxicity, human non-carcinogenic toxicity, mineral resource scarcity, fossil resource scarcity, land use, and water consumption (Table 4). In relation to global hot environmental issues, this study primarily focuses on 18 types of environmental impacts during the process of wastewater treatment and synchronous hydrogen production. The specific standardization results are shown in Table 5. The characterizing calculation results are obtained by multiplying the corresponding gas emissions with their respective relevant factors to obtain the final outcome [61].

After computing the characteristic values, it is necessary to standardize and assign weights to the obtained impact potentials. In this study, the environmental impact load of 1990 was taken as the benchmark value for weighted calculations, with a step value of 1. The formula for calculating standardized potential values of environmental pollutants’ effects is provided below [62,63]:

$${\mathrm{NPE}}_{m}={\mathrm{EP}}_{m}/{\mathrm{ER}}_{m}$$

In the provided equation, NPE*_m_* represents the normalized data value of the m-th type of environmental impact potential; EP*_m_* denotes the environmental potential value of the m-th type of environmental impact within the system; and ER*_m_* corresponds to the standardized data value of the m-th type of environmental impact potential. The computational outcome is depicted in Table 5.

### 3.3. Result Analysis and Improvement Measures

From the aforementioned data in Table 5, it can be inferred that the rank order of the negative impact on environmental types during the simultaneous hydrogen production process through photocatalytic treatment of waste is as follows: freshwater ecological toxicity > marine ecological toxicity > terrestrial ecological toxicity > non-carcinogenic toxicity to human beings > water eutrophication > terrestrial acidification > scarcity of mineral resources > terrestrial ecosystems > ozone formation, human health > global warming > carcinogenic toxicity to human beings > ionizing radiation > particle formation > water consumption > depletion of ozone layer > marine eutrophication > land utilization > scarcity of mineral resources. Freshwater ecotoxicity, marine ecotoxicity, terrestrial ecotoxicity, and human non-carcinogenic toxicity are the main environmental adverse effects in the process of hydrogen production from photocatalytic waste.

This study systematically analyzed the potential environmental impact of the simultaneous hydrogen production from 4-NP wastewater treated by CDs/CdS/CNU. According to the process flow of CDs/CdS/CNU treating 4-NP wastewater and producing hydrogen simultaneously, the primary source of these four toxicities is attributed to cadmium sulfide (CdS) present in the photocatalyst [40,64]. Although the inherent toxicity risk of CdS itself may be minimal, it possesses exceptional adsorption capacity and affinity, enabling it to adsorb pollutants in the environment. In aquatic ecosystems, due to the poor water solubility of CdS, it tends to accumulate and precipitate in sedimentary deposits within the water [65]. Consequently, CdS entering sediments has the potential to alter the ecological toxicity of pollutants within them, thus posing a threat to aquatic ecosystems. Additionally, as a result of some leaching issues with CdS catalysts, Cd can enter aquatic ecosystems and terrestrial ecosystems and ultimately impact human health. Due to its lack of essential biological functions, when it reaches a certain concentration within an organism, it will damage the biological cell membrane [66]. It has the capability to combine with sulfur-based groups (-SH), thus affecting the activity of various enzymes in the human body [67]. This, in turn, hampers normal physiological functions within cells and disrupts the DNA of living organisms. Furthermore, both the sediment and leaching metals from this aqueous substance can enter neighboring soil through treated water and be absorbed into the surrounding ecosystem by humans along its path. This causes toxic accumulation and subsequently results in ecological toxicity that disrupts the ecological equilibrium [68]. Hence, these are potential factors contributing to the occurrence of this environmental type. Although CdS has some environmental drawbacks as a photocatalyst, it is notable for its direct band gap semiconductor properties. CdS possesses a band gap of 2.4 eV, enabling it to absorb light with wavelengths shorter than 520 nm within sunlight. Additionally, its conduction band potential is -0.88 eV, and its valence band potential is 1.52 eV, which are suitable for hydrogen production through water reduction. As a direct band gap semiconductor, CdS also exhibits high photoelectric conversion efficiency and effective carrier diffusion, making it a prominent focus of research in photocatalytic hydrogen production [69,70].

Since the discovery of photocatalytic water decomposition in the 1970s, numerous semiconductor photocatalytic materials have been developed, including metal sulfides [71,72,73], metal oxides [74,75], metal oxynitrides [76,77], and organic semiconductors [78,79,80]. In the solar spectrum, ultraviolet light, visible light, and infrared light account for approximately 5%, 46%, and 49% of solar energy, respectively. To efficiently absorb sunlight over a broad wavelength range, photocatalytic materials generally require a narrow band gap. Under typical experimental pH conditions, the conduction band and valence band positions of most semiconductor compounds align well with the redox potential of water. However, these materials often have band gaps greater than 3.0 eV, limiting their excitation to ultraviolet light. In contrast, CdS exhibits a suitable band gap of approximately 2.4 eV and favorable energy band positions. Its absorption edge is at 520 nm, enabling strong responsiveness to the visible portion of the solar spectrum [69,70]. Moreover, the conduction band and valence band potential energies of CdS align with the requirements for photocatalytic water splitting (~1.23 eV) [81]. Consequently, CdS is considered an ideal catalyst for visible light-driven hydrogen evolution and presents significant potential for advancing the industrial application of photocatalysis. However, in addressing these environmental risks, one can endeavor to minimize the impact of environmental influencing factors and thereby alleviate cumulative environmental harm through the following measures. Addressing concerns regarding CdS’s significant adsorption affinity and capacity can be achieved through post-modification techniques aimed at morphologically altering CdS [82]. Transforming its structure into a hollow configuration enhances active sites and typically includes pores larger than pollutant dimensions, facilitating increased surface contact between catalysts and contaminants [83]. This enhancement leads to more efficient degradation of persistent toxic pollutants, thereby reducing their accumulation on the catalyst. Moreover, another cause of various toxicities lies in the leaching of cadmium (Cd) from CdS catalysts used in simultaneous hydrogen production during the photocatalytic treatment of waste [84]. Cd is a highly toxic non-biogenic heavy metal recognized as an environmentally hazardous substance due to its accumulative and latent properties, posing potential risks to terrestrial ecosystems and human health upon leaching [85]. To address the issue of metal leaching, subsequent measures can involve modifying the surface of CdS and grafting hydrophobic functional groups to stabilize the Cd-S bond [86]. This approach reduces its interaction with water, thereby mitigating the problem of metal leaching.

## 4. Conclusions

Wastewater treatment and simultaneous hydrogen production is one of the possible ways to solve environmental problems and the energy crisis, but its own environmental impact cannot be ignored. This study takes the synchronous hydrogen production process of 4-NP wastewater with CDs/CdS/CNU as a practical case to investigate the environmental impact of wastewater treatment and synchronous hydrogen production process throughout its life cycle. The results showed that the simultaneous treatment of wastewater and hydrogen production would have great potential effects on freshwater ecotoxicity, marine ecosystem toxicity, terrestrial ecotoxicity, and human non-carcinogenic toxicity. The freshwater ecotoxicological impact standard value during simultaneous hydrogen production and waste disposal is measured at 410.8, indicating that catalyst enrichment in waste disposal adversely affects freshwater ecosystems. Meanwhile, the marine ecotoxicological impact standard value stands at 274.5, suggesting that water body branching and confluence contribute to pollutant accumulation in marine ecosystems, thereby causing significant environmental repercussions. Through the analysis of the whole life cycle of the process, various ecotoxicological phenomena are derived from the adsorption and metal leaching characteristics of the catalyst during waste treatment and synchronous hydrogen production. Rational modification and morphology enhancement of these catalysts offer avenues to mitigate these impacts and enhance both waste disposal efficiency and simultaneous hydrogen production. Despite potential environmental pollution concerns inherent in waste treatment and simultaneous hydrogen production processes, effective waste management can mitigate these issues and present a viable solution to the energy crisis posed by hydrogen energy. This study demonstrates the feasibility of life cycle assessment in assessing the environmental impact of waste utilization technologies. The main potential environmental impacts of the synchronous hydrogen production process of 4-NP wastewater with CDs/CdS/CNU are proposed. It will provide a basis for the promotion and application of more wastewater treatment and synchronous hydrogen production technology, technical improvement, and government decision-making.

## Figures and Tables

**Table 3:** Emission of other gasses during the synchronous hydrogen production process of 4-NP wastewater treated with CDs/CdS/CNU.

Project	Detection 1	Detection 2	Detection 3	Detection 4	Detection 5	Average Value
Carbon dioxide (CO_2_) g·m^−3^	9450	9448	9449	9452	9448	9449.4
Sulfur dioxide (SO_2_) g·m^−3^	49	51	47	50	51	49.6
nitrous oxide (N_2_O) g·m^−3^	0	0	0	0	0	0
carbon monoxide (CO)g·m^−3^	5	5.5	5	5.8	5.6	5.38
nitrogen oxide (NO_x_)g·m^−3^	0.3	0.4	0.3	0.38	0.35	0.346
VOC g·m^−3^	225	220	223	228	221	223.4

**Table 4:** Characteristic value (synchronous hydrogen production process of 4-NP wastewater with CDs/CdS/CNU).

Impact Category	Unit	Altogether	Cadmium Sulfide	Phenylethane	Urea	Muriate	Citric Acid	Ammonia	Alcohol	Deionized Water	Power Consumption
**Global warming**	kg CO_2_ eq	23,340.50	0.60	45.20	82.74	0.43	5.74	1.72	329.9	0.024	22,871.2
**Stratospheric ozone depletion**	kg CFC11 eq	0.006	1.90 × 10^−7^	3.70 × 10^−6^	1.3 × 10^−4^	7.80 × 10^−8^	8.50 × 10^−6^	5.90 × 10^−7^	6.10 × 10^−5^	2.04 × 10^−8^	0.0062
**Ionizing radiation**	kBq Co-60 eq	326.34	0.06	0.67	4.67	0.02	0.13	0.02	10.96	0.001	309.82
**Human health**	kg NO_x_ eq	53.98	0.0018	0.071	0.16	0.0009	0.01	0.002	0.65	4.81 × 10^−5^	53.09
**Fine particulate matter formation**	kg PM2.5 eq	9.71	0.0004	0.023	0.052	0.0005	0.0029	0.001	0.11	1.60 × 10^−5^	9.52
**Land**	kg NO_x_ eq	54.15	0.002	0.08	0.16	0.0009	0.014	0.002	0.73	4.87 × 10^−5^	53.09
**Ground acidification**	kg SO_2_ eq	0.14	0.014	0.10	0.47	0.001	0.03	0.01	0.88	0.0001	136.24
**Eutrophication of freshwater.**	kg P eq	252.00	0.0003	0.009	0.03	9.60 × 10^−5^	0.002	0.0001	0.07	7.68 × 10^−6^	2.42
**Eutrophication of the sea**	kg N eq	0.21	1.70 × 10^−5^	0.0006	0.03	7.50 × 10^−6^	0.001	7.10 × 10^−6^	0.02	8.51 × 10^−7^	0.16
**Land ecological toxicity**	kg 1,4-DCB eq	6451.90	1.68	20.12	123.58	0.31	6.77	6.97	297.04	0.042	5995.37
**Freshwater ecological toxicity**	kg 1,4-DCB eq	417.53	0.06	0.50	4.65	0.01	0.26	0.03	6.19	0.017	405.83
**Seawater ecological toxicity**	kg 1,4-DCB eq	108.96	0.0167	0.17	1.27	0.003	0.07	0.01	2.07	0.0004	105.35
**Human carcinogenic toxicity**	kg 1,4-DCB eq	2.72	0.0002	0.049	0.082	7.50 × 10^−5^	0.0020	0.0013	0.12	1.15 × 10^−5^	2.46
**Non-carcinogenic toxicity in humans**	kg 1,4-DCB eq	125.67	0.025	0.38	2.17	0.005	0.14	0.023	4.86	0.0007	118.06
**Land use**	m^2^a crop eq	253.95	0.18	0.32	11.18	1.43	65.37	0.05	13.91	0.0033	161.49
**Scarcity of mineral resources.**	kg Cu eq	9.61	0.05	0.03	0.49	0.003	0.05	0.002	0.54	0.00045	8.45
**Scarcity of fossil resources**	kg oil eq	3277.05	0.18	20.67	22.47	0.071	0.99	0.55	152.46	0.0052	3079.64
**Water consumption**	m^3^	83.84	0.0065	0.48	1.51	0.0027	0.15	0.033	3.94	0.043	77.68

**Table 5:** Standardized value (synchronous hydrogen production process of 4-NP wastewater with CDs/CdS/CNU).

Impact Category	Altogether	Cadmium Sulfide	Phenylethane	Urea	Muriate	Citric Acid	Aqueous	Alcohol	Deionized Water	Power Consumption
**Global warming**	2.17	5.61 × 10^−5^	0.0042	0.00769	4.02 × 10^−5^	0.0005	0.0001	0.0306	2.3 × 10^−6^	2.13
**Ozone layer consumption**	0.098	2.88 × 10^−6^	5.71 × 10^−5^	0.00192	1.12 × 10^−6^	0.0001	8.99 × 10^−6^	0.0009	3.12 × 10^−7^	0.095
**Ionizing radiation**	0.695	0.0001	0.0014	0.0099	3.963 × 10^−5^	0.0002	4.42 × 10^−5^	0.0233	2.85 × 10^−6^	0.066
**Ozone formation, and human health**	2.621	8.63 × 10^−5^	0.003	0.0077	4.35 × 10^−5^	0.0006	9.44 × 10^−5^	0.0315	2.34 × 10^−6^	2.58
**Particulate matter formation**	0.606	2.65 × 10^−5^	0.001	0.0032	3.136 × 10^−5^	0.0001	5.98 × 10^−5^	0.0071	9.97 × 10^−7^	0.59
**Land ecology**	3.041	0.0001	0.0045	0.0092	5.102 × 10^−5^	0.0007	0.0001	0.0410	2.75 × 10^−6^	2.99
**Ground acidification**	3.360	0.0003	0.0024	0.0113	2.858 × 10^−5^	0.0007	0.0002	0.0215	2.89 × 10^−6^	3.32
**Water eutrophication**	3.880	0.0003	0.0138	0.0405	0.0001	0.0025	0.0001	0.1028	1.18 × 10^−5^	3.72
**Ocean eutrophication**	0.0457	3.77 × 10^−6^	0.0001	0.0057	1.62 × 10^−6^	0.0002	1.54 × 10^−6^	0.0048	1.85 × 10^−7^	0.034
**Land ecological toxicity**	11.613	0.0030	0.0362	0.2224	0.0005	0.0121	0.0125	0.5346	7.63 × 10^−5^	10.79
**Freshwater ecological toxicity**	410.8	0.05864254	0.4954	4.5725	0.0097	0.2529	0.0288	6.0905	0.0016	399.33
**Marine ecological toxicity**	274.5	93452	0.4316	3.19	0.0074	0.1811	0.0283	5.2045	0.0011	265.48
**Human carcinogenic toxicity**	1.167	0.0001	0.0210	0.0353	3.19 × 10^−5^	0.0008	0.0005	0.0527	4.93 × 10^−6^	1.06
**Non-carcinogenic toxicity in humans**	4.046	0.0007	0.0122	0.0698	0.0001	0.0045	0.0007	0.1563	2.28 × 10^−5^	3.80
**Land use**	0.041	2.94 × 10^−5^	5.16 × 10^−5^	0.0018	0.0002	0.0105	8.79	0.0022	5.29 × 10^−7^	0.03
**Scarcity of mineral resources.**	4.97 × 10^−5^	2.48 × 10^−7^	1.61 × 10^−7^	2.55E-06	1.54 × 10^−8^	2.46 × 10^−7^	1.13 × 10^−8^	2.78 × 10^−6^	2.33 × 10^−9^	4.38 × 10^−9^
**Scarcity of fossil resources**	3.34	0.0001	0.0210	0.0229	7.232 × 10^−5^	0.0010	0.0005	0.1555	5.35 × 10^−6^	3.14
**Water consumption**	0.31	2.45 × 10^−5^	0.002	0.0056	1.00 × 10^−5^	0.00055	0.00012	0.015	0.00016	0.29

## Data Availability

Data is contained within the article.
